# Criterion distances and correlates of active transportation to school in Belgian older adolescents

**DOI:** 10.1186/1479-5868-7-87

**Published:** 2010-12-08

**Authors:** Delfien Van Dyck, Ilse De Bourdeaudhuij, Greet Cardon, Benedicte Deforche

**Affiliations:** 1 Fund for Scientific Research Flanders (FWO), Brussels, Belgium; 2 Department of Movement and Sports Sciences, Ghent University, Ghent, Belgium; 3 Department of Human Biometrics and Biomechanics, Vrije Universiteit Brussels, Brussels, Belgium

## Abstract

**Background:**

Since physical activity levels in older adolescents have the potential to be increased by stimulating active transportation to school (ATS), the most important correlates of ATS should be determined before developing interventions, especially in those adolescents for whom the distance to school is feasible for active commuting. The main aims of this study were to determine criterion distances for ATS in Belgian older adolescents, to examine multidimensional correlates of ATS in adolescents living within a feasible distance from school and to investigate the associations of ATS with total physical activity and with other physical activities besides ATS.

**Methods:**

In total, 1281 older adolescents (17-18 years) from 20 general secondary schools in East- and West-Flanders completed a questionnaire on physical activity behaviors, demographic factors and psychosocial and physical environmental correlates of physical activity. Distance to school was objectively measured using Routenet online route planner.

**Results:**

In total, 58.4% of the participants commuted actively to school. The criterion distance for ATS could be set at eight kilometers for cycling and two kilometers for walking. For those adolescents living within a feasible distance for ATS, gender, smoking status, walkability of the neighborhood and social modeling were associated with transportation mode choice. ATS was positively associated with total physical activity, but not significantly related to min/week of other physical activities.

**Conclusions:**

For older adolescents living within eight kilometers of their school, interventions taking into account the correlates found to be related to ATS could possibly be effective to enhance ATS and to increase total physical activity levels. In the context of the overall physical activity decline in adolescence, also interventions targeting physical activity behaviors of adolescents living further away from school might be needed, but these interventions should probably emphasize alternative strategies.

## Background

In most developed nations, physical activity (PA) declines in adolescence with considerable proportions of adolescents not meeting the PA guideline of 60 minutes of moderate to vigorous PA daily [1]. To increase daily PA in adolescents, active transportation to school (i.e. walking and cycling to school; ATS) could be an important contributor [2]. Several studies in adolescents have shown that ATS is associated with higher overall moderate-to-vigorous PA [3,4], higher fitness levels [5,6] and higher daily energy expenditures [2] compared to passive commuting.

Nevertheless, before developing interventions enhancing ATS in adolescents, the most important multidimensional determinants of the target behavior need to be identified. Ecological models state that PA (including ATS) is influenced by an interplay between sociodemographic, psychosocial and physical environmental factors and that each of these factors needs attention in research [7,8].

Most research investigating correlates of ATS in adolescents only focused on young adolescents (12-16 years). For this age group, sociodemographic correlates of ATS are consistent with boys and low socio-economic status (SES) adolescents having higher ATS rates than girls and high-SES adolescents [8-13]. Some studies in young adolescents also investigated psychosocial correlates like social modeling, perceived barriers and attitude towards PA, but no significant associations with ATS have been found until now [9,10]. For neighborhood physical environmental factors, some evidence showed that living in urban areas [9], perceiving more land use mix [12] and perceiving higher connectivity [13] are related to more ATS in young adolescents, but the most consistent physical environmental correlate in this age group is distance to school. Many studies found distance to school to be negatively associated with ATS [9,12,14,15].

Research on ATS correlates in older adolescents (16-18 years) is much sparser. Only two studies in this age group were identified [16,17]. Nelson and colleagues [16] found that boys, adolescents living in densely populated areas and those living closer to school were more likely to do ATS, while Robertson-Wilson and colleagues [17] showed that boys, non-smoking adolescents and those attending an urban school were more likely to commute actively. Because limited evidence on the most important correlates of ATS in older adolescents exists, further research in this age group is necessary. Moreover, Belgian adolescents are allowed to drive a moped from the age of 16, possibly causing a decrease in ATS in older adolescents. Furthermore, since PA tends to track stronger from late adolescence to adulthood than from childhood or early adolescence to adulthood [18], enhancing ATS in older adolescence affect ATS in adult life positively. The transition from high school to university or college (at the age of 18 in Belgium) has also been shown to be a critical period for decreasing PA levels and weight gain [19,20]. Since 16-18 year old adolescents are supposed to undergo this transition in the near future, interventions increasing ATS in older adolescents might help to sustain the amount of active transportation after the transition.

As mentioned above, previous studies showed that distance to school was the most consistent physical environmental barrier to ATS in young adolescents [9,12,14-16]. Nonetheless, this distance barrier is very difficult to tackle since it is not possible to force adolescents to move closer to school or to choose a school closer to their home. However, a UK study in 15-17 year old adolescents showed that 40% of the adolescents commuting to school by car, lived within 4.0 km of their school, which might be a feasible distance for ATS. Consequently, investigating the multidimensional correlates of transport mode choice in adolescents for whom the distance to school is feasible for ATS, is important.

In that context, an important issue is the determination of a criterion distance within which ATS is feasible. According to a UK study in 15-17 year old adolescents, over 80% of the adolescents walking and cycling to school lived within respectively 2.4 km and 4.0 km from school [16]. Consequently, these distances were defined as criterion distances. Once distances to school exceeded these criteria, the percentage of adolescents commuting actively to school dropped clearly. To our knowledge, no other European studies have determined such criterion distances for ATS in adolescents. Since cycling rates are higher in Belgium than in England [21] and criterion distances can differ across age groups, it is recommended to determine country- and age-specific criterion distances.

Consequently, our main study aims were 1) to determine criterion distances for walking and cycling to school in Belgian older adolescents (17-18 years), 2) to examine sociodemographic, psychosocial and physical environmental correlates of ATS in those Belgian adolescents for whom the distance from home to school is feasible for active transportation, and 3) to investigate whether ATS is positively associated with total PA and with other PA besides ATS.

## Methods

### Participants and protocol

In total, 1281 adolescents (57.3% female, 17.1 ± 0.5 years) from 20 general secondary schools in East- and West-Flanders participated in the study. All adolescents attended the last year of secondary school. To recruit participants, 58 randomly selected schools were contacted by phone, of which 20 agreed to participate (response rate schools = 34.5%). Informed consent was obtained from school directors, parents and students. The study protocol was approved by the ethical committee of Ghent University Hospital. All participants completed a questionnaire on PA behaviors and correlates during classes, under supervision of a research assistant. Weight and height were objectively measured according to international standards [22].

### Measures

#### Demographic variables

Self-reported demographic variables included gender, age, living situation, living environment, smoking status, parental educational level, parental working status, home address and school address.

#### Psychosocial variables

All questions were derived from previous studies in adults and adolescents [23-25]. Social modeling, social norm, social support, self-efficacy and attitude towards PA were included. Social modeling was measured by asking how frequently family and friends participated in PA (two items, Cronbach's alpha (α) = 0.62). Social norm was assessed by asking if participants believed that family and friends wanted them to exercise (two items, α = 0.77). To investigate social support, participants were asked how often family and friends exercised together with them, invited them to exercise and encouraged them to participate in PA (six items, α = 0.74). The level of self-efficacy was obtained by asking how confident they were to be physically active under 14 potentially difficult situations (e.g. when tired, family expectations; α = 0.91). Attitude towards PA was calculated by subtracting the total perceived barriers towards PA (e.g. lack of time, lack of energy; 26 items, α = 0.94) from the total perceived benefits towards PA (e.g. enjoyment, weight loss; 18 items, α = 0.91). This calculation method follows the principles of the Health Belief Model [26], which explains that general attitude can be expressed as perceived benefits minus perceived barriers. All psychosocial correlates were rated on a 5-point Likert scale.

#### Perceived neighborhood physical environmental factors

To assess the perceived neighborhood environment, the Flemish NEWS questionnaire was used [27]. 'Neighborhood' was defined as 'the direct environment, everywhere within a 10-15 minute walk of your home'. Only those neighborhood physical environmental factors that have been shown to be consistently associated with active transportation in previous studies [28], were included in the analyses. Moreover, physical environmental factors being irrelevant for ATS, like access to recreational facilities and amount of home PA equipment, were not included in the analyses. Factors included were residential density, land use mix diversity and access, street network connectivity, availability and quality of walking and cycling infrastructures, safety for bicycle theft and perceived traffic safety.

The Flemish NEWS has acceptable to good reliability (intraclass correlation coefficients between .40 and .97) and acceptable validity (coefficients between .21 and .91) [27]. All environmental factors were rated on a 4-point scale, except for residential density (three-point) and land use mix diversity (5-point). Because of high intercorrelations (≥0.40) between residential density, land use mix diversity, land use mix access and connectivity, a 'walkability Z-score' was calculated based on the Z-scores of these four scales. This walkability score was based on the walkability formula of Frank and colleagues [29]: walkability = z-score residential density + 2*z-score connectivity + z-score land use mix. The walkability z-score was used in all analyses.

#### Physical activity and transportation mode to school

Physical activity was determined using a paper-and-pencil version of the Flemish Physical Activity Questionnaire, which has been validated in a computerized version [30]. In that questionnaire, transportation mode to school (walking, cycling, passive transportation) was queried. Passive transportation included transportation by car, moped and public transport. For some analyses, a dichotomized variable (active versus passive transportation) was constructed. Moreover, min/week of ATS, min/week of active transportation in leisure-time and min/week of leisure-time sports were assessed. Time spent in PA besides ATS (= other PA) was calculated by subtracting min/week of ATS from the total amount of PA (min/week). Total PA was obtained by summing up min/week of ATS, min/week of active transportation in leisure-time, min/week of leisure-time sports and min/week of sports at school.

#### Distance to school

Distance from the participants' home to school was objectively measured using Routenet online route planner http://www.routenet.be, taking into account both school and home address data. The shortest route from home to school was used.

### Data analysis

Descriptive statistics and criterion distances for walking and cycling to school were analyzed using SPSS 16.0. To determine criterion distances for ATS, cumulative percentages of participants commuting actively to school per covered distance (ranges of 1km) were examined. Criterion distances were set at a distance within which approximately 85% of the active commuters lived.

Multilevel regression analyses were conducted using MLwiN version 2.10. Multilevel modeling (two-level: participant-school) was applied to take into account clustering of paticipants within schools. To examine associations between the independent variables (sociodemographics, physical environmental perceptions, psychosocial factors) and transportation mode choice (active/passive transportation: dummy variable), two-level logistic regressions were used. Odds ratios with confidence interval (95%) are given for each independent variable. To investigate associations of transportation mode choice (independent variable: dummy) and min/week of ATS (independent variable) with total PA (dependent variable) and with the amount of other PA (dependent variable), two-level linear regression models were constructed. These analyses were controlled for the significant correlates of transportation mode choice. For all analyses, significance was set at p ≤ 0.05.

## Results

### Sample characteristics

Sociodemographic characteristics, as well as mean scores of the psychosocial and physical environmental variables are shown in Table 1. Of the total sample, 6.6% walked to school, 51.8% cycled to school and 41.5% used passive transportation. Mean overall distance from home to school was 6.57 (6.17) km. For the adolescents walking or cycling to school, mean distance was 1.31 (1.03) km and 4.80 (2.87) km respectively. For the passive commuters, mean distance 9.74 (8.04) km.

**Table 1:** Descriptive characteristics of the sample

Variable	Total sample (n = 1281)
**Sociodemographic characteristics**	
Gender (%)	
Male	42.7
Female	57.3
Age (mean (SD))	17.14 (0.51)
Living situation (%)	
Living with both parents	80.5
Not living with both parents	19.5
Living environment (%)	
Coastal environment	0.4
Countryside	18.6
Village	42.5
(Sub)urban environment	38.6
Educational level mother (%)	
No college/university degree	32.8
College/university degree	67.2
Educational level father (%)	
No college/university degree	35
College/university degree	65
Working status mother (%)	
Not working	13
Working	87
Working status father (%)	
Not working	3
Working	97
Smoking status (%)	
Non-smoker	88.9
Smoker	11.1
Body Mass Index in kg/m^2 ^(mean (SD))	
Boys	21.84 (2.96)
Girls	21.60 (2.84)
**Psychosocial factors**	
Social modeling	3.35 (0.78)
Social norm	3.18 (1.10)
Social support	2.51 (0.74)
Self-efficacy	3.44 (0.86)
Attitude towards PA	1.32 (1.13)
**Neighborhood physical environmental perceptions**	
Walkability Z-score	0.01 (2.89)
Walking infrastructure	2.74 (0.61)
Cycling infrastructure	2.34 (0.56)
Safety for cycling	2.43 (0.40)
Traffic safety	2.74 (0.49)
**Mode of transportation to school (%)**	
Walking	6.6
Cycling	51.8
Passive (car, public transit, motor)	41.5
**Distance to school in km (mean (SD))**	
Overall	6.57 (6.17)
Walking	1.31 (1.03)
Cycling	4.80 (2.87)
Passive transportation (car, public transit, motor)	9.74 (8.04)

SD = standard deviation

### Criterion distance for different transportation modes

Table 2 shows the (cumulative) percentages of participants walking, cycling and commuting passively to school per covered distance. In total, 83.5% of the adolescents walking to school lived within 2.0 kilometers from school. The other 16.5% lived within 5.0 kilometers. Of the adolescents who cycled to school, 85.6% lived within 8.0 kilometers from school and 94% within 10 kilometers. Approximately 44% of the passive commuters also lived within 8.0 kilometers from school.

**Table 2:** Distance covered by transportation mode

Distance (km)	Walking (n = 85)		Cycling (n = 664)		Passive transport (n = 532)	
	%	Cum%	%	Cum%	%	Cum%
0.1 - 1.0	50.6	50.6	3.8	3.8	0.2	0.2
1.1 - 2.0	32.9	**83.5**	13.2	17.0	1.2	1.4
2.1 - 3.0	8.9	92.4	1.81	35.0	4.7	6.1
3.1 - 4.0	2.5	94.9	12.2	47.2	4.7	10.8
4.1 - 5.0	5.1	100.0	12.5	59.7	7.4	18.2
5.1 - 6.0			9.5	69.3	8.8	27.0
6.1 - 7.0			8.1	77.7	8.0	35.0
7.1 - 8.0			8.2	**85.6**	9.2	**44.2**
8.1 - 9.0			6.2	91.8	9.2	53.4
9.1 - 10.0			2.2	94.0	8.4	61.8
> 10.0			6.0	100.0	38.2	100.0

km = kilometer

Cum% = cumulative percent

**Figure 1:**
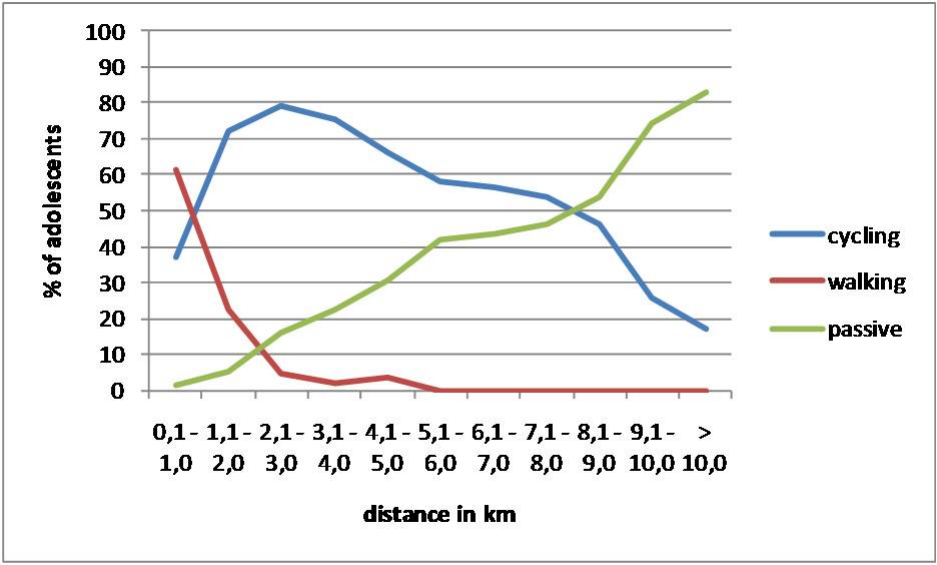
**Percentage of adolescents per transportation mode for each covered distance**.

The results shown in Figure 1 confirm these findings. In that figure, the percentages of cyclists, walkers and passive commuters living at each distance from school are presented. For walking, a clear breaking point in the curve can be observed at 2.0 km. For cycling, the breaking point is less clear, but at a distance of 8.1 - 9.0 km from home to school, the cycling curve crosses the passive commuting curve. At that distance, the percentage of passive commuters becomes higher (53.6%) than the percentage of cyclists (46.4%).

### Correlates of transportation mode choice

Based on the results from Table 2 and Figure 1 criterion distances could be set at two kilometers for walking and eight kilometers for cycling. Consequently, to examine correlates of transportation mode choice in adolescents for whom distance to school is feasible for ATS, only participants living within 8 kilometers from school were included in the regression analyses (i.e. 888 adolescents).

The logistic regression analyses (Table 3) revealed that gender, smoking status, walkability of the neighborhood and social modeling were significantly associated with transportation mode choice. Girls (OR = 0.58; 95% CI = 0.41-0.84) and smokers (OR = 0.61; 95% CI = 0.41-0.92) were less likely to do ATS than boys and non-smokers. Moreover, adolescents perceiving higher neighborhood walkability (OR = 1.10; 95% CI = 1.02-1.17) and more social modeling (OR = 1.23; 95% CI = 1.01-1.51) were more likely to do ATS than those perceiving lower walkability and less social modeling. For the other sociodemographic, physical environmental and psychosocial factors, no significant results were found.

**Table 3:** Logistic multi-level analyses of sociodemographic, physical environmental and psychosocial correlates of transportation mode to school

Dependent variable: Transportation mode to school: 0 = passive transportation, 1 = active transportation			
Predictor	β (SE)	Odds ratio	95% CI
**Gender (ref: male)**	**-0.539 (0.184)**	**0.58**	0.41, 0.84*
Educational attainment mother (ref: no college/univ)	-0.176 (0.190)	0.84	0.58, 1.21
Educational attainment father (ref: no college/univ)	0.146 (0.187)	1.16	0.80, 1.67
**Smoking status (ref: non-smoker)**	**-0.488 (0.207)**	**0.61**	0.41, 0.92*
Body Mass Index	0.028 (0.028)	1.03	0.97, 1.09
**Walkability Z-score**	**0.091 (0.033)**	**1.10**	1.02, 1.17*
Walking infrastructure	0.127 (0.173)	1.14	0.81, 1.60
Cycling infrastructure	-0.024 (0.176)	0.98	0.69, 1.38
Safety for bicycle theft	0.232 (0.212)	1.26	0.83, 1.91
Traffic safety	0.117 (0.175)	1.12	0.80, 1.58
**Social modeling**	**0.211 (0.102)**	**1.23**	1.01, 1.51*
Social norm	-0.016 (0.088)	0.98	0.83, 1.17
Social support	0.089 (0.134)	1.09	0.84, 1.42
Self-efficacy	0.033 (0.130)	1.03	0.80, 1.33
Attitude towards PA	0.111 (0.098)	1.12	0.92, 1.35

SE = standard error, 95% CI = 95% confidence interval, PA = physical activity

* p < 0.05

### Associations of transport mode choice, min/week of active transportation to school with total PA and other PA

For these analyses, two linear regression models were constructed. In the first model, the associations of transport mode choice (active versus passive) with total PA and with other PA were examined. This model showed that, after controlling for the significant correlates of transport mode choice (i.e. gender, smoking status, walkability score and social modeling), active commuting to school was positively associated with total PA (β = 0.206, SE = 0.014, p < 0.001), but not significantly associated with min/week of other PA (β = 0.033, SE = 0.037, p = n.s.).

In the second model, the associations of the amount of ATS (in min/week) with total PA and with other PA were investigated. Results showed that, after controlling for the same correlates, min/week of ATS was positively related to total PA (β = 0.106, SE = 0.006, p < 0.001), but not significantly associated with min/week of other PA (β = 0.008, SE = 0.017, p = n.s.).

## Discussion

Our results showed that 58.4% of the adolescents commuted actively to school. Of these active commuters, 88.7% cycled to school and 11.3% walked to school. These percentages are higher than those found in the UK and Canada, where respectively 37.5% and 42.5% of the 15-18 year olds engaged in ATS [16,17]. Nevertheless, a Portuguese study in 13-18 year old adolescents found that 66.3% of the participants commuted actively to school, which is higher than the percentages found in this study [31]. In contrast with our findings, these other studies reported a much higher prevalence of walking to school (85.8% to 92.1% of the active commuters) than cycling to school (7.9 to 14.2%). The high prevalence of cycling to school found in our 17-18 year old participants could possibly be contributed to the flat landscape, long cycling tradition and cycling-friendly infrastructures in Belgium, resulting in high cycling rates in all age groups [[21,32], Cardon et al, unpublished observations]. Moreover, in Belgium a driver's license (for cars and motorbikes) can only be obtained from the age of 18 years, so adolescents in the last year of secondary school are not yet allowed to drive to school.

The results of the first research question showed that the criterion distance for ATS in older Belgian adolescents could be set at eight kilometers for cycling (85% of the cyclists lived within this distance from school) and two kilometers for walking (83.5% of the walkers lived within this distance). Nevertheless, the number of adolescents walking to school was limited, so further research is necessary to confirm this two kilometer criterion distance. To our knowledge, only one other study determined criterion distances in this age group [16]. In that study, criterion distances were 2.4 km for walking and 4.0 km for cycling. The criterion for walking is comparable between the two studies, but for cycling a greater distance was found in our study. This difference could also be due to the high cycling rates, cycling-oriented culture, flat landscape and cycling-friendly infrastructures in Belgium.

Nonetheless, more than 40% of the adolescents living within eight kilometers from school commuted passively to school. These adolescents are important targets for behavior change, since distance to school should be no major barrier. When focusing on those adolescents living within eight kilometers, gender, smoking status, social modeling and perceived neighborhood walkability were associated with transport mode choice. Other studies in young adolescents also found that boys and non-smoking adolescents were more likely to commute actively than girls and smokers [4,9,10,17]. Consequently, future interventions could focus specifically on these at-risk groups. Especially for smoking adolescents, a multibehavioral intervention targeting both PA and smoking behavior could be relevant. In contrast with other studies in young adolescents [11,13], parental education did not contribute to the likelihood of ATS. This might be due to a lack of variety in educational levels in our sample. Because only adolescents attending general secondary schools were included, educational levels were rather high. Nevertheless, when comparing the high and low SES adolescents (32.5% of the participants' mothers had a low educational level [33]), no differences were found for transport mode choice (χ^2 ^= 0.54, p = n.s.). This is promising, suggesting that interventions targeting ATS might be effective for both high and low SES older adolescents.

Concerning the psychosocial correlates, only PA levels of family and friends were positively associated with ATS. If future studies can confirm this result, it would give added value to involve the social environment (family and friends) of adolescents in interventions to increase ATS. The few other studies that included psychosocial factors did not find any significant associations with ATS in young adolescents [9,10]. A possible explanation for this limited evidence could be that active transportation is rather a habitual behavior than as a conscious choice, based on psychosocial constructs [34]. So, a supportive physical environment might possibly be more important to stimulate ATS than psychosocial factors.

Adolescents perceiving high neighborhood walkability were more likely to do ATS. Other studies including street connectivity [10,13], population density [16] or land use mix [35] as a potential correlate of adolescents' transport mode choice, found similar results. However, one Belgian study comparing PA of 12-18 year old adolescents living in a high-walkable town centre with PA of adolescents living in a less-walkable suburb, found similar percentages of active commuters in both neighborhoods [36]. This inconsistency across Belgian studies could possibly be explained by the age difference across the samples and the different methods used to measure walkability (perceptions versus objective measures). Because in general, study results on the importance of walkability features to encourage ATS in children and adolescents are still inconclusive [36-38], further research is recommended. Furthermore, walkability characteristics of school environments and the route to school should be included in future studies, since not only the neighborhood around their home, but also the school environment is important in explaining adolescents' PA [39]. Perceptions of availability and quality of walking and cycling infrastructures, safety for bicycle theft and traffic safety did not contribute significantly to the likelihood of ATS in this study. Especially traffic safety might be of higher importance in children and younger adolescents, since other studies showed that parental perceptions of safety are crucial in the decision about a child's travel mode [40,41].

The results of the last research question showed that active (versus passive) transportation to school and min/week of ATS were positively associated with total PA, but not with other PA besides ATS. These findings suggest that the higher amounts of total PA in active commuters were caused by ATS only. Many other studies have also found a positive association between ATS and total PA [2-4,42] but mixed results have been found for the association between ATS and other PA. A Portuguese study showed that ATS was positively related to non-organized other PA among 13-16 year old boys, but not among girls [31] and Landsberg and colleagues [4] found similar gender-specific results in 14-year-old German adolescents. A study in Filipino youth supported our results and found that the higher amount of total energy expenditure in active commuters was due to active commuting only [2]. When interpreting our results and the findings of Tudor-Locke and colleagues [2], it is positive that adolescents doing ATS appear not to compensate this healthy behavior with less PA in other domains. Moreover, the finding that ATS was not associated with other PA suggests that not only those adolescents who already engage in many leisure-time PA commute actively. This is promising for future interventions, because as long as the distance is feasible, ATS could be stimulated in both active and less active older adolescents. However, more research is needed to confirm our findings. Future research should also address possible associations between ATS, total PA and health parameters, since no consensus on the health effects of ATS is reached yet [4,31,43].

Strengths of this study firstly include the large study sample. Secondly, the study was executed in 17-18 year old adolescents. Almost no other studies have investigated the ATS correlates in this age group, and since the transition from high school to university has been shown to be a critical period to decrease PA [19,20], research in this age group is important. Thirdly, validated questionnaires were used. Moreover, distance to school was measured objectively. Some study limitations also need to be acknowledged. First, only self-reported PA was assessed. Future studies should consider including objective PA measures, since self-report measures possibly suffer from social desirability and over-reporting [24]. Second, the questions on psychosocial correlates did not specify a type or domain of PA, so it might be that the participants tended to consider mainly leisure-time PA when responding. This lack of specificity might have influenced the present results. Third, only adolescents attending general secondary schools were included, limiting the generalizability of the results. Fourth, only the main transport mode of the adolescents was captured in the questionnaire. Probably, some adolescents used mixed transportation (e.g. combination bicycle - public transit), which is healthier than using only passive transportation. Fifth, in the questionnaire, passive transportation was not specified in terms of car use, public transit or moped use. For other research questions, this information would be interesting. Sixth, no characteristics of the school environment (physical environment, school policies, encouragement of ATS,...) and of the route to school were included in this study, while these factors are probably associated with the amount of ATS. Seventh, the online routeplanner did not take off-street facilities and cut-throughs into account; only streets were considered when calculating distances from home to school. Consequently, actual walking and cycling distances might be shorter than the distances calculated using the online routeplanner.

## Conclusions

In conclusion, the criterion distance for ATS in older Belgian adolescents was set at 8 kilometers in this study. For adolescents living within this distance of their school, gender, smoking status, social modeling and the perception of walkability contributed significantly to the likelihood of ATS. If other studies can confirm these findings, researchers should take these factors into account when developing interventions to enhance ATS and total PA in older adolescents. Of course, in the context of the overall physical activity decline in adolescence, interventions targeting PA behaviors of adolescents living further than eight kilometers from school might also be needed, but these interventions should probably emphasize alternative strategies to increase overall PA.

## Competing interests

The authors declare that they have no competing interests.

## Authors' contributions

All authors contributed to the design of different parts of the study. DVD coordinated the data collection, conducted the statistical analyses and drafted the manuscript. BD developed the data collection protocol and coordinated the data collection. BD, GC and IDB participated in the interpretation of the data, helped to draft the manuscript and revised the manuscript for important intellectual content. All authors read and approved the final manuscript.
